# The SoyaGen Project: Putting Genomics to Work for Soybean Breeders

**DOI:** 10.3389/fpls.2022.887553

**Published:** 2022-04-26

**Authors:** François Belzile, Martine Jean, Davoud Torkamaneh, Aurélie Tardivel, Marc-André Lemay, Chiheb Boudhrioua, Geneviève Arsenault-Labrecque, Chloe Dussault-Benoit, Amandine Lebreton, Maxime de Ronne, Vanessa Tremblay, Caroline Labbé, Louise O’Donoughue, Vincent-Thomas Boucher St-Amour, Tanya Copley, Eric Fortier, Dave T. Ste-Croix, Benjamin Mimee, Elroy Cober, Istvan Rajcan, Tom Warkentin, Éric Gagnon, Sylvain Legay, Jérôme Auclair, Richard Bélanger

**Affiliations:** ^1^Département de Phytologie, Université Laval, Quebec City, QC, Canada; ^2^Centre de Recherche sur les Grains (CEROM), Saint-Mathieu-de-Beloeil, QC, Canada; ^3^Agriculture and Agri-Food Canada, St-Jean-sur-Richelieu, QC, Canada; ^4^Agriculture and Agri-Food Canada, Ottawa, ON, Canada; ^5^Department of Plant Agriculture, University of Guelph, Guelph, ON, Canada; ^6^Department of Plant Sciences, University of Saskatchewan, Saskatoon, SK, Canada; ^7^Semences Prograin Inc., Saint-Césaire, QC, Canada; ^8^Sevita Genetics, Inkerman, ON, Canada; ^9^Sollio Agriculture, Saint-Hyacinthe, QC, Canada

**Keywords:** translational genomics, genetic diversity, marker-trait associations, haplotypes, genomic prediction

## Abstract

The SoyaGen project was a collaborative endeavor involving Canadian soybean researchers and breeders from academia and the private sector as well as international collaborators. Its aims were to develop genomics-derived solutions to real-world challenges faced by breeders. Based on the needs expressed by the stakeholders, the research efforts were focused on maximizing realized yield through optimization of maturity and improved disease resistance. The main deliverables related to molecular breeding in soybean will be reviewed here. These include: (1) SNP datasets capturing the genetic diversity within cultivated soybean (both within a worldwide collection of > 1,000 soybean accessions and a subset of 102 short-season accessions (MG0 and earlier) directly relevant to this group); (2) SNP markers for selecting favorable alleles at key maturity genes as well as loci associated with increased resistance to key pathogens and pests (*Phytophthora sojae*, *Heterodera glycines*, *Sclerotinia sclerotiorum*); (3) diagnostic tools to facilitate the identification and mapping of specific pathotypes of *P. sojae*; and (4) a genomic prediction approach to identify the most promising combinations of parents. As a result of this fruitful collaboration, breeders have gained new tools and approaches to implement molecular, genomics-informed breeding strategies. We believe these tools and approaches are broadly applicable to soybean breeding efforts around the world.

## Introduction

In the last 50 years, plant genetics has entered into the age of molecular biology and recombinant DNA. The main benefits of these technological advances in the development of improved varieties have come in the form of DNA markers to assist in the identification of breeding lines with a specific desired attribute (through marker-assisted selection, MAS) and in the development of transgenic plants (genetically modified organisms, GMOs), sometimes providing novel traits that could not be introduced through crosses. In both cases, prior to any sort of application, a discovery phase is needed through which a gene/QTL is found to confer or contribute a desirable attribute. One common means to discovering such genes/QTLs is through genetic mapping. It has often been argued that, despite the astounding number of QTL mapping studies conducted in crop species, only a fairly limited subset of these has led to the development of DNA markers that are actually used in breeding programs (Bernardo, 2016).

More recently, advances in two additional areas have contributed to the breeder’s toolkit and brought new promises: next-generation sequencing (NGS; Goodwin et al., 2016; Mahmoud et al., 2019) and gene-editing technologies (Knott and Doudna, 2018; Anzalone et al., 2019). The increasing availability and decreasing cost of NGS technologies have opened up a new era in crop genomics where genetic diversity can be extensively captured both in the form of numerous high-quality genome assemblies and pangenomes for soybean (Liu Y. C. et al., 2020; Bayer et al., 2021; Torkamaneh et al., 2021) as well as in large collections of re-sequenced lines (Liu Y. C. et al., 2020; Torkamaneh et al., 2020). In addition, NGS technologies have allowed major strides to be made in the use of genome-wide association studies (GWAS) to identify genomic regions and candidate genes of interest to breeders (Gupta, 2021). For their part, gene-editing technologies are providing unique opportunities to directly obtain desired allelic variants in elite genetic backgrounds and are allowing for the functional validation of numerous candidate genes initially identified via GWAS (Kumlehn et al., 2018).

Breeding programs are in a unique position to benefit from these advances. As eventual practitioners of MAS, breeders have a keen interest in ensuring that markers relevant to their breeding objectives are developed. Fortunately, the mapping of the underlying genetic determinants of a trait relies on the genetic and phenotypic characterization of various collections of individuals, be they the progeny of a controlled cross as in biparental QTL mapping or unrelated individuals in the case of association mapping. The generation and characterization of such populations require expertise that is generally present in modern-day breeding programs. These mapping populations can either be a “side product” of the breeding activity *per se* or constitute a “side project” that naturally builds on the capabilities of a breeding program.

A few hundred polymorphic markers, if well distributed, provide sufficient coverage to perform biparental QTL mapping, thanks to the extremely limited amount of recombination that has occurred in populations of F_2_ or recombinant inbred lines (RILs). Such relatively low numbers of markers can easily be obtained using various relatively low-cost genotyping technologies such as genotyping-by-sequencing (GBS; Sonah et al., 2013) or a SNP array such as the SoySNP6K (Song et al., 2020). In contrast, dense genome-wide coverage with SNP markers is a pre-requisite for a successful GWAS as many more recombination events are captured in collections of unrelated lines, thus leading to a much lower amount of linkage disequilibrium (LD) between markers. By its nature, GWAS is particularly attractive to breeding programs as it can provide highly linked markers for traits that are present with the germplasm of interest to a program.

It is with the aim of putting genomics to work for the benefit of breeding programs that the SoyaGen project was funded by Genome Canada (along with a host of other co-funders; see extensive list in see “Funding” section below) in the context of its Large-Scale Applied Research Projects (LSARP) program. A team of genomicists, geneticists, breeders, pathologists, and social scientists was assembled to overcome key challenges faced by the soybean crop in Canada. In the breeding realm, these revolved around maximizing realized yield through varieties offering optimal maturity and increased disease/pest resistance. At the end of the project (June 2021), the SoyaGen team has achieved, and in some cases exceeded, many of the goals that it had set for itself and many of these advances are widely relevant to soybean breeders interested in making greater use of DNA markers and genomic information.

## Main Achievements of the Soyagen Project

### Genome-Wide Marker Coverage to Explore and Characterize Existing Genetic Diversity

**FIGURE 1:**
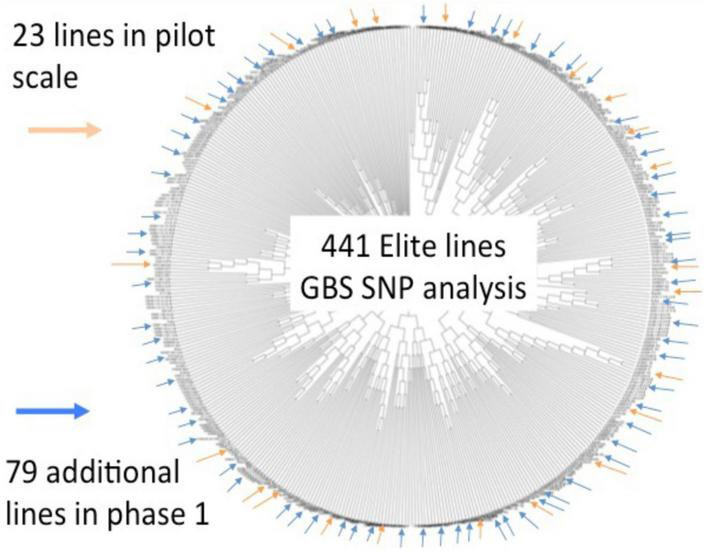
Summary of whole-genome sequencing work done on a collection of short-season soybean breeding germplasm from Canada. An initial genotypic characterization of a collection of 441 lines from three breeding programs was performed using GBS (∼50K SNPs). Based on a tree capturing the genetic relationships between these lines, a subset of 102 lines was selected in view of whole-genome sequencing. This resulted in a catalog of close to 5M SNPs and small indels as well as close to 100K structural variants.

An initial set of 441 soybean accessions (i.e., breeding lines and cultivars) contributed by three public breeding programs (University of Guelph, Agriculture and Agri-Food Canada-Ottawa, CÉROM) were genotyped via GBS, resulting in a set of ∼50,000 informative SNPs. These data were used to capture the genetic relatedness between these lines and a representative subset of 102 lines was selected to undergo whole-genome sequencing (WGS) (Figure 1). As detailed in Torkamaneh et al. (2017), this resulted in a set of close to 5M SNPs and small indels as well as close to 100K structural variants (>50 bp in size) that comprehensively described genetic variation within short-season soybean in Canada. Importantly, such exhaustive marker coverage provides the ability to precisely define SNP haplotypes across the genome. On a global level, haplotypes can be extremely useful tools for the imputation of missing data. On a local level, haplotypes can capture the allelic state at a gene of interest. Both of these were exploited in the course of SoyaGen.

The collection of 102 lines subjected to WGS was used as a reference panel for the imputation of SNP genotypes at missing loci. Starting with a SNP dataset derived from low-cost GBS (530 lines, 150K SNPs), Torkamaneh et al. (2017) demonstrated that it was possible to use this reference panel as a basis to perform genome-wide imputation. In essence, the number of genotyped SNP markers was increased from 150K to almost 5M simply by imputing the genotypes at missing loci. The accuracy of the resulting dataset was found to be in excess of 96%. Such dense and accurate SNP data can then be used to perform high-resolution GWAS as exemplified in Boudhrioua et al. (2020), Bruce et al. (2020), Malle et al. (2020), and Seck et al. (2020) for resistance to Sclerotinia stem rot, for amino acid content in seed, root-system architecture as well as yield and agronomic traits, respectively.

**FIGURE 2:**
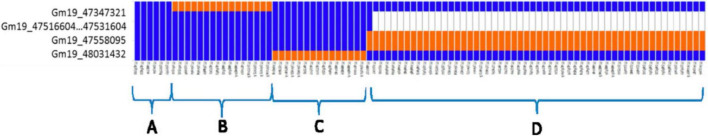
Graphical representation of single nucleotide polymorphism (SNP) haplotypes for 91 early maturing accessions. Each vertical bar corresponds to one individual, each horizontal line corresponds to one SNP marker. Blue represents the allele present in the reference genome (Williams 82) and orange the alternate allele. White is used to indicate an absence of reads mapping in the *E3* (*GmPhyA3*) gene within a 13-kb segment that is deleted in the *e3-tr* allele. Joint consideration of these polymorphisms allowed the identification of four distinct haplotypes (A–D). Reproduced with permission from Tardivel et al. (2014).

Another extremely powerful use for such dense marker coverage is in the discovery and genotyping of specific alleles at loci of interest. As one can imagine, in the short summers experienced in Canada, it is critical to develop and use varieties carrying alleles that confer earliness at key maturity genes. Given that there can be many alleles for a single gene, it follows that no single biallelic SNP can adequately capture this diversity. Using dense sets of markers (initially from GBS alone), Tardivel et al. (2014) demonstrated that SNP haplotypes could precisely identify known alleles at the *E3* maturity locus (Figure 2) and allowed the identification of a novel allele that had not yet been reported (*E/e3p.Thr832Ala*). Once millions of SNPs became available, through WGS and imputation (as described above), it then became possible to define haplotypes and allelic variants at four key maturity loci (*E1-E4*; Tardivel et al., 2019). Defining haplotypes among such large sets of SNPs did not prove trivial, however, and it required the development of a tool capable of sifting through these data to extract those SNPs that were most informative. This tool, HaplotypeMiner (described in Tardivel et al., 2019), essentially translates a large amount of genotypic information (in the form of SNP data) into more useful catalogs of alleles at these loci.

**FIGURE 3:**
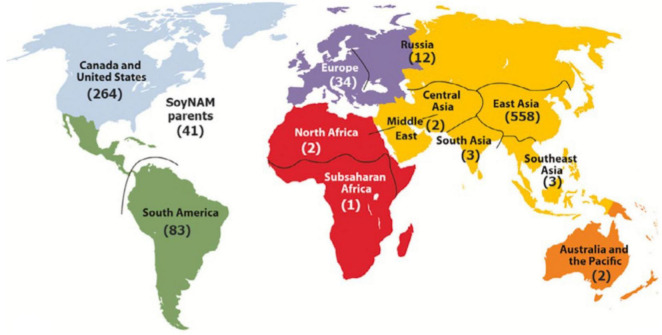
Geographical distribution of GmHapMap accessions. Reproduced with permission from Torkamaneh et al. (2020).

To broaden the contributions of the SoyaGen team to the worldwide soybean community, the tools and know-how acquired through this work were also used to provide soybean geneticists and breeders with a key resource for translational and functional genomics. The first soybean haplotype map (GmHapMap) was produced within the context of the SoyaGen project, with help from international collaborators (Torkamaneh et al., 2020). In brief, WGS data (some novel and some already in the public domain) were collected for a set of 1,007 worldwide soybean accessions (Figure 3) and allowed the identification of close to 15M SNPs and small indels. It was demonstrated that this collection of lines provided extensive coverage of the nucleotide diversity in *Glycine max*.

**FIGURE 4:**
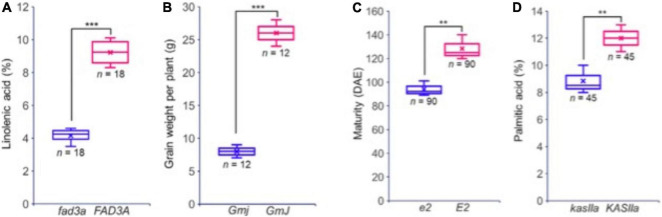
Phenotypic variation observed between accessions with (blue) and without (red) a predicted LOF mutation in four different genes. **(A)** *FAD3A*, a key gene for linolenic acid synthesis; **(B)** *GmJ*, a key gene for the Long Juvenile trait; **(C)** *GmGIa* (*E2*), a key gene controlling maturity; **(D)**, *KASIIa*, a key gene in the oil biosynthesis pathway. In each case, the number of accessions sharing the same allele (and for which phenotypic data were at hand) is indicated. Reproduced with permission from Torkamaneh et al. (2020). ** means that *p* ≤ 0.05, *** means that *p* ≤ 0.01.

Building on this data, two reference panels, one including only genic SNPs and the other all SNPs, were produced and can be used to perform extensive imputation in cultivated soybean. The HaplotypeMiner tool was then used on this dataset to identify SNP haplotypes in and around each of the ∼55K soybean genes. In an illustration of how this can be of use to breeders, the allele present at the *E2* locus controlling maturity was defined for each of the over 1,000 accessions. Finally, using software to predict the functional impact of SNPs located within coding regions, a total of 18,031 variants predicted to cause a loss of function (LOF) were identified. These were found to reside in 10,662 genes, representing approximately 20% of all soybean genes. It was demonstrated that lines carrying an LOF allele in a gene exhibited an altered phenotype compared to lines containing a functional copy of the gene (Figure 4). This constitutes an extremely valuable tool allowing breeders to explore the allelic and functional variants present within the accessions of the GmHapMap collection. Most importantly, all of these resources are in the public domain and can be readily accessed via a dedicated page on the SoyBase web site.^1^

Finally, the set of WGS data available for the 1,007 accessions of the GmHapMap was used to select a subset of 204 phylogenetically and geographically representative accessions and to produce a pan-genome for cultivated soybean, PanSoy (Torkamaneh et al., 2021). This allowed us to uncover 108 Mb of novel sequence that was absent from the Williams82 reference genome. Within these novel sequence contigs, over 3,600 protein-coding genes (including 1,659 novel genes) were found. Nonetheless, globally over 90% of soybean genes were shared by > 99% of the sequenced accessions of *G. max*, indicating a very large and highly conserved core genome.

### Selection Tools for Maturity and Disease Resistance

As was described above, dense SNP genotyping facilitated the identification of SNP markers capable of tagging the various alleles found at the known loci controlling maturity (reviewed recently in Lin et al., 2020). For the *E1* to *E4* genes, the SNP haplotypes among the breeding germplasm were used to infer the specific allelic makeup of the soybean accessions at these four loci (Tardivel et al., 2014, 2019). Thus, when breeders design crosses with the objective of increased earliness, early parental lines that differ in their allelic makeup at these loci can be crossed with the expectation that transgressive segregants will be obtained.

As it was known that a there are additional genes that control maturity, beyond the four described above that had been cloned at the onset of this work, a GWAS was performed to identify further genes controlling maturity among a panel of 86 PIs belonging to maturity groups 00 and 000 (Copley et al., 2018). In addition to already known *E* genes, a novel association was detected on chromosome13 near a trio of orthologs of the Arabidopsis *HAP5* gene, one shown to promote flowering under long days (Cai et al., 2007; Kumimoto et al., 2008). Altogether, a suite of 18 allele-specific PCR markers (mostly KASP) were developed and are routinely used in marker-assisted selection.

**FIGURE 5:**
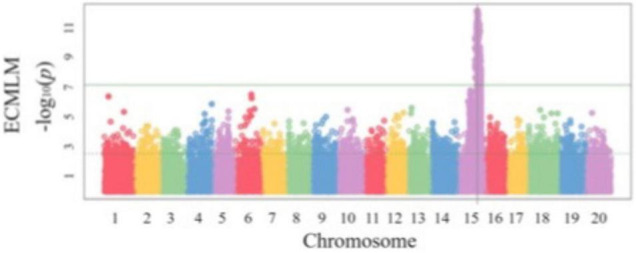
Genome-wide association mapping of resistance to *Phytophthora sojae* in a soybean population of 357 plant introductions (PIs). Reproduced with permission from de Ronne et al. (2021).

Another important part of SoyaGen team’s efforts aimed to develop selection tools for increased resistance toward three particular pathogens/pests: (1) *Phytophthora sojae*, the causal agent of Phytophthora root rot (PRR), *Heterodera glycines*, the soybean cyst nematode (SCN) and *Sclerotinia sclerotiorum*, the cause of Sclerotinia stem rot (SSR) or white mold. These had been identified as the three threats of greatest concern to soybean growers in Canada. In the case of *P. sojae*, two types of resistance are known (Dorrance, 2018). Vertical resistance confers immunity to specific pathotypes of *P. sojae* on the basis of a gene-for-gene interaction between a single *Rps* gene in the host and an *Avr* gene in the pathogen (Lebreton et al., 2018). Horizontal resistance, on the other hand, confers a broad but incomplete protection against all pathotypes of *P. sojae*. In the SoyaGen project, much of the QTL mapping work on resistance to *P. sojae* was aimed at identifying genomic regions contributing to horizontal resistance. In a first study, a biparental QTL mapping approach was used to investigate the genetic determinants of partial horizontal resistance in PI449459 (de Ronne et al., 2019). Two QTL (one each on Gm13 and Gm19), each explaining approximately 15% of the phenotypic variance for *P. sojae* resistance, were identified and SNP markers associated with these loci became available to select for these favorable alleles. A GWAS approach was also used to investigate horizontal resistance among a large panel of 357 fully re-sequenced soybean lines (de Ronne et al., 2021). Interestingly, within this panel, a very strong association (FDR-adjusted *p*-value = 4.8 × 10^–7^) was detected on Gm15 (Figure 5). As the allele contributing to reduced PRR severity is shared by more than 60 lines, it provides a large set of potential sources of this allele as well as tightly associated SNP markers. The SNP markers associated with these QTL offer a unique opportunity to stack resistance loci using MAS and to complement the resistance conferred by *Rps* genes when the latter is ineffective.

Similarly, in the case of SCN, efforts were focused on the discovery of QTL associated with horizontal resistance. QTL mapping was performed within the progeny of a biparental cross derived from PI494182, an accession reported to confer partial resistance against multiple HG types of SCN (Young, 1995; Arelli and Wang, 2008). Following GBS genotyping and testing of resistance against an SCN population of HG type 0, six QTL were identified (Boucher St-Amour et al., 2020). Interestingly, in addition to the known *Rhg-1* (Gm18) and *Rhg-4* (Gm08) loci (Patil et al., 2019), one of the other QTL (Gm11) mapped near the *GmSNAP11* gene, one which has also been implicated in SCN resistance (Lakhssassi et al., 2017). Thus, through this work, new SNP markers were gained to complement the previous MAS work focused on selecting desirable alleles at the *Rhg-1* and *Rhg-4* loci.

### Diagnostic Tools to Facilitate the Identification and Mapping of Specific Pathotypes of *Phytophthora sojae*

One important challenge that breeders face when trying to develop varieties with genetic resistance to important pests or pathogens is that genes that confer vertical resistance are only effective against a specific subset of pathotypes. For this reason, it is essential to have information on the predominant pathotypes (specific allelic makeup at relevant *Avr* genes) that are present within a field or a larger cropping area to allow informed decisions to be made regarding the resistance gene(s) to deploy to confer effective protection. In the case of *P. sojae*, a pathogen estimated to cause yearly losses of $50M in Canada, and over $500M in the United States (Sepiol et al., 2017), five *Rps* genes are most commonly deployed commercially (*Rps1a*, *1c*, *1k*, *3*, and *6*; Dorrance, 2018) but their efficacy can be limited by the large number of described pathotypes (Dorrance et al., 2004). Surveys of the pathotypes found in growers’ fields are typically performed using the hypocotyl inoculation test developed by Kaufmann and Gerdemann (1958) on a set of differential lines. Unfortunately, this method is time-consuming, laborious and prone to false-positives and -negatives.

**FIGURE 6:**
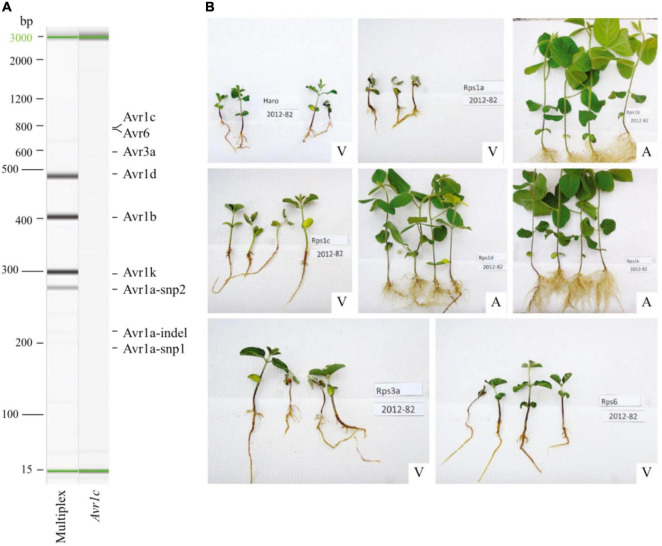
Comparison of molecular and phenotyping assays to determine the pathotypes of *Phytophthora sojae* isolates. **(A)** Gel image of multiplex polymerase chain reaction (PCR) amplifications of discriminant regions associated with avirulence alleles for seven *Avr* genes in *P. sojae* isolate 2012–82. Presence of amplicons for *Avr1b*, *1d*, and *1k* predicts a pathotype 1a, 1c, 3a, and 6. **(B)** Phenotyping results for isolate 2012–82 indicates a compatible interaction with Harosoy (*rps*), *Rps1a*, *Rps1c*, *Rps3a*, and *Rps6* and an incompatible interaction with *Rps1b*, *Rps1d*, and *Rps1k*, thereby assessing a pathotype 1a, 1c, 3a, and 6, similar to the molecular assay. A, avirulent and V, virulent. Reproduced with permission from Dussault-Benoit et al. (2020).

To overcome these limitations, Arsenault-Labrecque et al. (2018) performed whole-genome sequencing on a selected set of 31 *P. sojae* isolates covering the spectrum of pathotypes found in Canada using a more reliable hydroponic inoculation test that closely reflects the natural course of infection (Lebreton et al., 2018). It became possible to identify haplotypes at seven *Avr* genes (*Avr1a, 1b, 1c, 1d, 1k, 3a, 6*) and SNP markers associated with virulence and avirulence, as assessed on differential lines harboring a specific *Rps* gene. Based on this in-depth knowledge of allelic variants, Dussault-Benoit et al. (2020) developed a multiplex PCR assay to identify *P. sojae* pathotypes based on the detection of specific alleles at the *Avr* genes (Figure 6). This test proved much more rapid as it could yield a result in a matter of hours (starting from DNA of an isolate of unknown pathotype), instead of weeks with the hypocotyl test, and was highly accurate as it matched the result of the phenotyping (hydroponic assay) in over 97% of cases.

**FIGURE 7:**
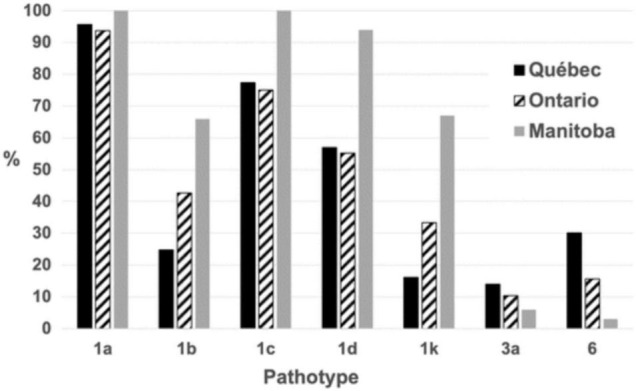
Percentage of *Phytophthora sojae* isolates carrying a given pathotype. The percentage is based on 295 isolates of *P*. *sojae* recovered in Québec, Ontario, and Manitoba fields in 2018 and 2019. Reproduced with permission from Tremblay et al. (2021).

With these tools in hand, it then became possible to perform field surveys on a broader scale. As reported recently by Tremblay et al. (2021), a characterization of close to 300 isolates, derived from the main soybean-growing areas in Canada (provinces of Ontario, Quebec and Manitoba), revealed 31 different pathotypes. Importantly, this survey indicated that *Rps1a* and *1c* were no longer effective in controlling *P. sojae* in Canada as 98 and 86%, respectively, of isolates carried alleles allowing them to overcome these resistance genes (Figure 7). On a national level, *Rps3a*, and *Rps6* provided the greatest degree of efficacy against the pathotypes found. These results suggest that a number of the currently deployed *Rps* genes are no longer effective, that a select few retain efficacy and that a diversification of resistance genes (*Rps* or horizontal resistance QTL) would be desirable. In addition, it was found that 85% of growers used varieties susceptible to *P. sojae* isolates found in their fields. Such information is highly useful to breeders to guide decisions on the introgression of genes/QTL conferring resistance to *P. sojae*.

Similarly, the overuse of SCN-resistant lines, mostly derived from a single parental germplasm (PI 88788) has led to the multiplication of virulent SCN populations (Mitchum, 2016). As for *P. sojae*, the development of rapid diagnostic tools for HG types would be a great asset to inform breeders on the prevalence and distribution of virulence alleles in SCN populations. An important step toward this goal has recently been accomplished by Ste-Croix et al. (2021) by identifying SCN transcripts whose abundance is associated with the ability to overcome the resistance conferred by PI 88788 and Peking, the main resistant parental lines used by breeders.

### Genomic Prediction to Identify the Most Promising Combinations of Parents

In a breeding program, the selection of parents to use in crosses is quite challenging. Even when a breeder identifies a selected subset of lines that he/she wishes to use as parents, the number of possible crosses can often widely exceed the number of crosses that can be made and whose progeny can be tested. Even a modest set of only 30 candidate parental lines generates close to 900 potential crosses (if considering both parents as male and female). It would be of interest to breeders if genomic information could be used to help guide some of these decisions.

**FIGURE 8:**
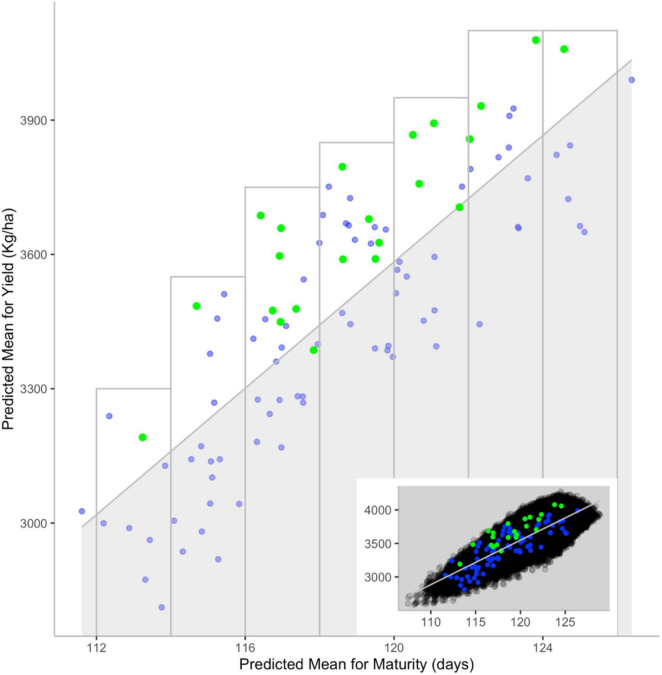
Comparison between predicted values for yield and maturity and persistence during selection. The main scatterplot shows the correlation between predicted progeny mean for yield (*y*-axis) and maturity (*x*-axis) for validation (blue) and superior (green) crosses. Inset scatterplot showing how values from the main graph are distributed among those from other cross sets: All (black), validation (blue) and superior (green) crosses. The shaded area contains crosses with predicted below-average yield for a given maturity [i.e., crosses below the correlation line (gray)]. The gray rectangles showcase crosses with above-average yield for a given maturity. Reproduced with permission from Jean et al. (2021).

To explore this question, Jean et al. (2021) used genotypic and phenotypic information on a set of 350 lines to predict the mean performance of over 60,000 potential crosses for yield and maturity, two key traits of prime concern to soybean breeders. To assess the accuracy of these predictions, a subset of 101 crosses that had been performed and subjected to selection in the course of past breeding work was examined. A superior cross was deemed one in which at least one derived breeding line was entered into registration trials or was commercialized. Interestingly, of the 22 superior crosses among this set, over 90% (20/22) had been predicted to offer above-average yield within a specific maturity window (Figure 8). Conversely, over 96% of crosses predicted to exhibit below-average yield (again, within incremental maturity windows) had been eliminated in the course of selection. These results suggest that it is possible to guide breeders’ choice of the most promising parental combinations using the genomic makeup of candidate parental lines.

## Conclusion

The last few years have seen exciting developments in the application of genomic technologies to breeding. In our view, genomic approaches offer a radically new opportunity to provide breeders with much more relevant information. The dense marker coverage achievable through NGS makes it possible to much more accurately capture allelic diversity at loci of interest. Whereas SNP markers have traditionally been limited to discriminating between two alleles at a single locus, the transition toward a more global view of the SNP landscape at a locus of interest, through which it is possible to define haplotypes, offers much more meaningful and useful information to breeders. As was illustrated above, using SNP markers in the vicinity of known maturity genes makes it possible to readily determine the allelic makeup of any soybean line. For a breeder, knowledge of the specific allele(s) present at loci of interest is much more relevant than a multitude of SNP data. Designing crosses between pairs of early parental lines, in view of obtaining transgressive segregation, is greatly facilitated when the allelic makeup of the parents is known to differ and to offer the opportunity for recombination to produce even earlier progeny. In addition, it is now possible to precisely assess how different combinations of alleles at such loci perform under different environmental conditions. In future, one can also imagine using powerful new gene editing technologies to generate a desired allelic variant in a suitable genetic background (Liu S. L. et al., 2020).

Similarly, the SNP haplotypes in and around *Avr* genes in *P. sojae* allowed the identification of haplotypes associated with virulence or avirulence toward a specific *Rps* gene in soybean. As a result, it became possible to characterize the pathotypes of *P. sojae* isolates through a simple multiplex PCR diagnostic assay. In the context of numerous plant pathogens, such an ability to map the specific pathotypes of a pathogen that are present in a field or in a cropping region provides extremely valuable information to breeders. Currently, soybean breeders typically introgress a single *Rps* gene in the varieties they develop in the hope that this gene will prove effective. Too often, unfortunately, this gene may no longer provide the desired resistance, as was illustrated for the *Rps1a* and *Rps1c* genes in the major soybean cropping areas of Canada.

Finally, beyond individual genes that a breeder may wish to introduce or maintain during cultivar development, for many an important trait such as yield, a focus on one or a few genes is simply impossible because of the polygenic nature of such traits. In this context, using a genomic prediction approach was shown to be a promising tool to assist the breeder in designing a crossing block. In theory, one could choose to select only parental combinations predicted to generate superior progeny or, alternatively, use genomic prediction to filter a list of potential crosses to eliminate those predicted to yield poorly. The first scenario is not without risk as these “superior” crosses, identified for two traits considered (e.g., yield and maturity), might not combine well with other desired traits. In the second scenario, it is fairly obvious that crosses offering inferior yield (within a given maturity window) will under no circumstances be viewed as promising. In the data shown above, many of the crosses that were made by the breeders, despite their extensive experience and informed judgment, were nonetheless predicted to produce progeny with below-average yield, thus making them unlikely to lead to improved varieties. The ability to filter in such a way a list of potential crosses could allow a breeding program to make the same genetic gains while considerably reducing the number of crosses, hence resources needed. Alternatively, maintaining the same research effort (number of crosses), while ensuring that all or most retained crosses offer a chance at selecting superior progeny, could lead to increased genetic gains per breeding cycle.

Through close collaboration between researchers with a broad range of expertise, and thanks to a focus on real-world problems facing breeders on a daily basis, the SoyaGen project has demonstrated that genomic tools have much to offer to the plant breeding community. Such a model for collaborative research is one which we feel could be replicated and help breeders address important challenges that are upon them with regards to a need for increasing agricultural productivity in the face of a changing climate.

## Author Contributions

FB and RB were project co-leaders and oversaw the entire project as well as leading activities focused on genetic diversity and genotyping (FB), as well as disease diagnostics and markers for resistance (RB). LO’D was activity leader for the group working on maturity and yield. All other authors directly contributed to the described work by designing or performing experiments, contributing to data analysis or supervising the work of students, postdocs, and research assistants involved in this work. All authors contributed to the article and approved the submitted version.

## Conflict of Interest

SL and ÉG were employed by the Semences Prograin Inc. ÉG was employed by the Sevita Genetics. JA was employed by the Sollio Agriculture. The remaining authors declare that the research was conducted in the absence of any commercial or financial relationships that could be construed as a potential conflict of interest.

## Publisher’s Note

All claims expressed in this article are solely those of the authors and do not necessarily represent those of their affiliated organizations, or those of the publisher, the editors and the reviewers. Any product that may be evaluated in this article, or claim that may be made by its manufacturer, is not guaranteed or endorsed by the publisher.

## Footnotes

1
https://www.soybase.org/projects/SoyBase.C2020.01.php
