# Supplementary material for: The SoyaGen Project: Putting Genomics to Work for Soybean Breeders
Source: Front Plant Sci. 2022 Apr 26;13:887553. doi: 10.3389/fpls.2022.887553 (PMC9087807; doi:10.3389/fpls.2022.887553)
Supplement: Supplementary file 1 [file Table_1.docx]

| Data type | Description | Repository | Accession/Link |
| --- | --- | --- | --- |
| WGS G. max | 102 elite Canadian cultivars  181 PIs and international cultivars | SRA-NCBI  SRA-NCBI | SRP094720/ <https://www.ncbi.nlm.nih.gov/sra/?term=SRP094720>  SRP318486/ <https://www.ncbi.nlm.nih.gov/sra/?term=SRP318486> |
|  |  |  |  |
| WGS P. sojae | 31 Canadian isolates of *P. sojae* | SRA-NCBI | SRP154202/ <https://www.ncbi.nlm.nih.gov/sra/?term=SRP154202> |
|  |  |  |  |
| RNA-seq G. max | *P. sojae*-infected soybean roots | SRA-NCBI | SRP223582/ <https://www.ncbi.nlm.nih.gov/sra/?term=SRP223582> |
|  |  |  |  |
| GmHapMap | SNPs/Indels/Haplotypes/Loss-of-function (LOF) alleles for 1,007 worldwide *G. max* accessions | SoyBase | <https://soybase.org/projects/SoyBase.C2020.01.php> |
|  |  |  |  |
| PanSoy | Pan-genome based on WGS data for 204 accessions of *G. max* (including predicted genes and presence/absence variants) | SoyBase | <https://soybase.org/projects/SoyBase.C2021.01.php> |

Supplementary Table 1. Main datasets generated during the SoyaGen project. A short description of the data and a link to the data are provided.
